# A pilot study of *Bifidobacterium breve* in neonates undergoing surgery for congenital heart disease

**DOI:** 10.1186/2052-0492-2-36

**Published:** 2014-06-05

**Authors:** Takako Umenai, Nobuaki Shime, Takashi Asahara, Koji Nomoto, Toshiyuki Itoi

**Affiliations:** 1 Department of Anesthesiology, Shimada Hospital, Osaka 583-0875, Japan; 2 Department of Anesthesiology and Intensive Care, Postgraduate School of Medical Science, Kyoto Prefectural University of Medicine, Kyoto 602-8566, Japan; 3 Department of Emergency and Critical Care Medicine, National Hospital Organization Kyoto Medical Center, Kyoto 612-8555, Japan; 4 Yakult Central Institute for Microbiological Research, Tokyo 186-8650, Japan; 5 Department of Pediatric Cardiology and Nephrology, Postgraduate School of Medical Science, Kyoto Prefectural University of Medicine, Kyoto 602-8566, Japan

**Keywords:** Probiotics, Neonates, Congenital heart surgery, Intestinal microbiota

## Abstract

**Background:**

Probiotics have currently been widely used in patients undergoing various types of surgeries and improved their clinical outcomes, while data in pediatric cardiac surgery have been lacking. We investigated the safety and effects on the intestinal microbiota of the probiotic *Bifidobacterium breve* in neonates undergoing surgery for congenital heart disease.

**Methods:**

This pilot, randomized study was performed in a single-center, university hospital-based pediatric intensive care unit (PICU). Twenty-one neonates undergoing surgery for congenital heart disease at >7 days after birth were randomly allocated to two groups: group A received 3 × 10^9^ colony-forming units (CFU)/day of enteral *B. breve* strain Yakult (BBG-01), which was started 1 week before and terminated 1 week after surgery (*n* = 10), and group B did not receive BBG-01 (*n* = 11).

**Results:**

The characteristics of the patients were similar in both groups. The postoperative days until fulfillment of the criteria for discharge from the PICU tended to be fewer in group A (8 [7–8] days) than in group B (9 [8–14] days) (*p* = 0.10). Likewise, the postoperative days to enteral nutrition or achievement of caloric goal tended to be fewer in group A than in group B. The *Bifidobacterium* in fecal samples after initiating BBG-01 in group A were significantly higher in number than that in group B. *Enterobacteriaceae* were significantly fewer in group A than in group B immediately (7.0 [3.9–7.7] vs. 8.5 [8.0–9.1] log_10_ cells/g) and 1 week (7.7 [7.0–8.1] vs. 9.3 [8.6–9.5] log_10_ cells/g) after surgery (*p* < 0.05 for both comparisons). The number of *Pseudomonas* after 1 week was significantly lower in group A than in group B (*p* = 0.04). The concentrations of total organic and acetic acids were also significantly higher in group A than in group B. The postoperative course was uncomplicated and all neonates were discharged alive from the PICU.

**Conclusions:**

The perioperative administration of a probiotic to neonates undergoing surgery for congenital heart disease was safe and significantly improved their intestinal environment. The positive effects of this treatment on clinically significant outcomes remain to be investigated.

## Background

Surgery for congenital heart diseases is performed in neonates worldwide with high success rates [1]. Infections and acute organ failure remain important complications and causes of reoperation, prolonged hospitalization, and intensive care, which significantly increase the postoperative morbidity and mortality [2,3]. Neonates undergoing corrective or palliative cardiac surgery are at increased risk of mesenteric hypoxia due to oxygen desaturation and low cardiac output. These patients often receive antimicrobials for prophylactic or non-prophylactic indications. Moreover, the incidence of delayed enteral feeding due to respiratory or cardiovascular instability is high [4]. These factors, alone or in combination, may disrupt the intestinal microbiota and function of the intestinal barrier, followed by bacterial translocation and associated disorders [5,6].

Probiotics are live microorganisms that confer benefits to a host when administered in sufficient amounts [7]. They have been widely used in critically ill [8,9] or cancerous patients undergoing surgery [10,11] and allegedly improved their clinical outcomes. The beneficial effects are likely due to an enhanced immune response to pathogens, competition for nutrition with pathogenic bacteria, improved immunologic function of the intestinal barrier, and a downregulation of proinflammatory cytokines [12,13].

Despite the putative therapeutic effects they confer to neonates at risk of intestinal failure, studies of probiotics in neonates undergoing neonatal surgery for congenital heart disease are scarce [14]. Therefore, in this pilot study, we examined the effects of the perioperative administration of probiotic bacteria, *Bifidobacterium breve*[15-17], on the intestinal microbiota and the clinical outcomes of neonates who underwent cardiac surgery.

## Methods

### Patients

**Figure 1:**
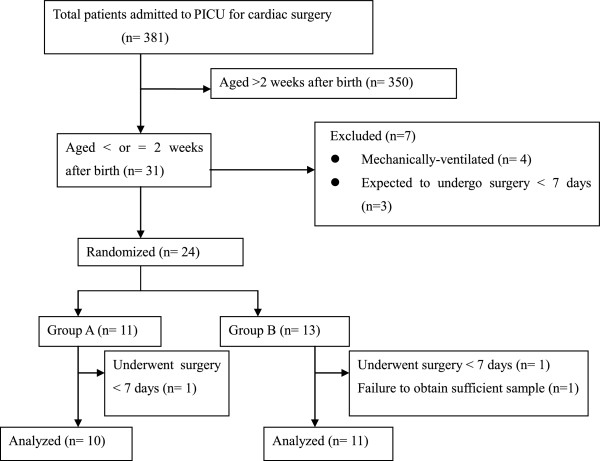
**Flow diagram.** A total of 21 patients were finally analyzed. *PICU* pediatric intensive care unit.

This study was approved by the ethics committee for clinical investigation of Kyoto Prefectural University, School of Medicine (Kyoto, Japan). Between April 2007 and April 2010, neonates admitted to our pediatric intensive care unit (PICU) and scheduled to undergo cardiac surgery between 1 to 2 weeks after birth were enrolled in this study. After excluding patients who were expected to undergo surgery within 7 days (*n* = 3) or who had received mechanical ventilation (*n* = 4), we enrolled 24 patients for randomized analysis. Of those, data from 21 patients who actually underwent surgery after over 7 days of age were finally analyzed (Figure 1). A signed informed consent was obtained from their parents. The patients were randomly assigned to group A, who received 3 × 10^9^ colony-forming units (CFU)/day of enteral *B. breve* strain Yakult (BBG-01), which was administered starting 1 week before and ending 1 week after surgery (*n* = 10), and group B, who did not receive BBG-01 (*n* = 11). BBG-01 was a live, freeze-dried probiotic, containing 10^9^ bacteria/g, and was generously provided by Yakult Central Institute for Microbiological Research under a written agreement. We chose the dose on the basis of previous pediatric studies, where BBG-01 was administered in doses ranging between 1 × 10^9^[15] and 4 × 10^9^ CFU/day [17].

All patients received prophylactic intravenous cefazolin, 25 mg/kg every 8 h, for 24 h, started immediately before the surgical incision. Enteral breast or artificial milk was fed upon the decision of the attending physicians, beginning with 1–2 ml/kg every 3 h, and increased by 2 ml/kg with each feeding to a target of 8 ml/kg every 3 h, if the gastric residuals were <50% of the administered dose and in the absence of vomiting or diarrhea.

### Clinical endpoints

We measured the rates of postoperative infections and the survival rate at the time of discharge from the PICU. We also counted the postoperative days until (a) the criteria for discharge from the PICU were fulfilled, (b) initiation of or candidacy for milk feeding, and (c) spontaneous defecation. The criteria for discharge from the PICU were the observation of stable vital signs and freedom from all indwelling lines, including the pericardial drains.

### Fecal bacteriological examination

#### Sample collection

Fecal samples were collected (1) before the administration of BBG-01, (2) immediately before the operation, 1 week after the start of BBG-01 administration, (3) immediately after surgery, and (4) 1 week after the operation. For the sampling of the postoperative samples, we allowed delay <48 h in cases of lacking defecation. Immediately after defecation, the samples were weighed and suspended in nine volumes of RNAlater® (Ambion Inc., Austin, TX, USA), an RNA stabilization solution, and then incubated for 10 min at room temperature.

#### Isolation of total RNA or total DNA

For RNA or DNA stabilization, 200 μl of fecal homogenate was added to 1 ml of sterilized phosphate buffer solution and then centrifuged at 5,000 *g* for 10 min. The supernatant was discarded and the pellet was stored at −80°C until used for the extraction of RNA or DNA. Methods described elsewhere were used to isolate RNA [18] and DNA [19]. Finally, the nucleic acid fraction was suspended in 1 ml of nuclease-free water (Ambion Inc.).

#### Bacterial count

A standard curve was generated using reverse transcription-quantitative polymerase chain reaction (RT-qPCR) data, using the threshold cycle, the number of cycles when threshold fluorescence was reached, and the corresponding cell count, which was determined microscopically with 4,6-diamidino-2-phenylindole staining (Vector Laboratories, Burlingame, CA, USA) for the dilution series of the standard strains described elsewhere [18]. To determine the number of bacteria present in the samples, three serial dilutions of an extracted RNA sample were used for the RT-qPCR, and the threshold cycle values in the linear range of the assay were applied to the standard curve generated in the same experiment, to obtain the corresponding bacterial cell count in each nucleic acid sample, which was then converted to the number of bacteria per sample. The specificity of the RT-qPCR assay using group- or species-specific primers was determined as described previously [18]. The quantitative analysis of *B. breve* strain Yakult has been described elsewhere [19]. The detection limits were as follows: 5.0 cells/g for the *Clostridium coccoides* group, *Clostridium leptum* subgroup, *Bacteroides fragilis* group, *Bifidobacterium*, *Atopobium* cluster, and *Prevotella*, 2.1 cells/g for *Clostridium perfringens*, 2.1 cells/g for total *Lactobacillus*, 3.9 cells/g for *Enterobacteriaceae*, 3.9 cells/g for *Enterococcus*, 3.6 cells/g for *Staphylococcus*, 2.9 cells/g for *Pseudomonas*, and 6.0 cells/g for *B. breve* strain Yakult. We treated data below the detection limit as each detection limit for statistic analysis.

### Fecal organic acid concentrations and pH

A sample of the homogenized stool was isolated, weighed, mixed with 0.15 M perchloric acid in a fourfold volume, and reacted for 12 h at 4°C. The mixture was centrifuged at 20,000 *g* for 10 min at 4°C, and the supernatant was filtered with a 0.45-μm membrane filter (Millipore Japan, Tokyo, Japan) and sterilized. The concentration of organic acids in the sample was measured using a Waters high-performance liquid chromatography system and 432 Conductivity Detector (Waters Co., Tokyo, Japan) and a Shodex Rspack KC-811 column (Showa Denko, Tokyo, Japan) [11,16]. We prepared a standard mixed solution containing 1 to 20 mM of succinic, lactic, formic, acetic, propionic, isobutyric, butyric, isovaleric, and valeric acids and calculated the concentrations of these organic acids based on the standard curve. The stool pH was measured by inserting the glass electrode of a D-51 pH meter (Horiba Seisakusho, Tokyo, Japan) directly into the samples of homogenized stool. The detection limits were as follows: 0.075 μmol/g for succinic acid, 0.2 μmol/g for lactic acid, 0.05 μmol/g for formic acid, 0.4 μmol/g for acetic acid, 0.5 μmol/g for propionic acid, 0.55 μmol/g for butyric acid, 0.8 μmol/g for isovaleric acid, and 0.65 μmol/g for valeric acid. We treated data below the detection limit as each detection limit for statistic analysis.

### Statistics analysis

The results are expressed as median [interquartile range (IQR)]. Mann-Whitney’s *U* test was used to compare between two groups, and comparison of the time-dependent data between two groups was performed using Wilcoxon’s signed-rank test.

## Results

The mean weights, gender distributions, and risk-adjusted congenital heart surgery (RACHS) category were similar, while the numbers of cardiac deformity involving aortic arch anomalies tended to be greater in group B (Table 1). No patient suffered complications from the probiotic administration.

**Table 1:** Patient characteristics and congenital diseases

	**Group A**	**Group B**
	**(*n*** **= 10)**	**(*n*** **= 11)**
Weight, kg	2.9 [2.8–3.0]	3.0 [2.9–3.1]
Males	5 (50%)	8 (73%)
Procedural times, min		
Overall operation	340 [267–378]	300 [220–332]
Cardiopulmonary bypass	152 [138–200]	167 [82–190]
Anesthesia	475 [395–503]	400 [298–428]
RACHS category	4 [3–4]	4 [3–4]
Congenital anomalies		
Transposition of great vessels	5	4
Interruption of the aortic arch	1	3
Double outlet right ventricle	2	0
Hypoplastic left heart syndrome	0	2
Coarctation of the aorta	2	1
Total anomalous pulmonary venous return	0	1
Selected types of surgeries		
Arterial switch	6	4
Aortic arch repair	3	4
Pulmonary arterial banding	1	1
Norwood surgery	0	1
Total anomalous pulmonary venous return repair	0	1

*RACHS* risk-adjusted congenital heart surgery. Values are median [IQR] or numbers (%) of observations. No significant differences between the groups.

### Clinical endpoints

No patient died or developed necrotizing enterocolitis. One patient in group B developed an infection at the surgical site. The criteria for postoperative discharge from the PICU tended to be fulfilled earlier (8 [7–8] days) in group A than in group B (9 [8–14] days) (*p* = 0.10; Table 2). Likewise, the mean time to (a) initiation of or candidacy for enteral nutrition and (b) spontaneous defecation tended to be shorter in group A than in group B (Table 2).

**Table 2:** Clinical endpoints

	**Group A**	**Group B**	***p *** **value**
	**(*n*** **= 10)**	**(*n*** **= 11)**	
Duration of MV, h	44 [18–67]	42 [19–37]	0.62
Days until			
Discharge criteria fulfilled, days	8 [7–8]	9 [8–14]	0.10
Target enteral nutrition, days	5 [4–6]	6 [5–8]	0.09
Spontaneous defecation, days	2 [1–2]	2 [2–5]	0.09

*MV* mechanical ventilation. Values are median [IQR].

### Fecal microbiota

No significant differences in total numbers of fecal microbiota were observed between the two groups (Table 3). The number of *Bifidobacterium* was significantly higher in group A than in group B throughout the courses. BBG-01, the probiotic administered, was isolated from the feces in group A only (Table 3). Concordantly, the number of *Enterobacteriaceae* in the log_10_ scale was significantly lower in group A than in group B, both immediately (7.0 [3.9–7.7] vs. 8.5 [8.0–9.1]) and 1 week (7.7 [7.0–8.1] vs. 9.3 [8.6–9.5]) after surgery (*p* < 0.05 for both comparisons). The number of *Staphylococcus* 1 week after surgery in the log_10_ scale was also significantly lower in group A than in group B (Table 3). The number of *Pseudomonas* after 1 week was significantly lower in group A than in group B (*p* = 0.04).

**Table 3:** Fecal microorganisms

			**Surgery**					
	**Before BBG**		**Before**		**Immediately after**		**1 week after**	
	**Group A**	**Group B**	**Group A**	**Group B**	**Group A**	**Group B**	**Group A**	**Group B**
	***n*** **= 7**		***n*** **= 10**	***n*** **= 11**	***n*** **= 10**	***n*** **= 11**	***n*** **= 10**	***n*** **= 11**
Total	9.7 [8.9–9.9] (100)	NT	9.8 [9.1–10.2] (100)	9.7 [9.3–9.9] (100)	9.9 [9.5–10.6]**** (100)	9.8 [8.7–10.0] (100)	9.9 [9.5–10.1] (100)	9.8 [9.5–9.9] (100)
Obligate anaerobes								
*Clostridium coccoides* group	8.7 [8.6–8.8] (29)	NT	6.4 [6.2–7.2] (30)	7.3 [6.2–8.4] (18)	7.9 [7.3–8.4] (40)	8.6 [8.1–8.6] (27)	6.2 [6.0–6.7] (50)	8.4 [7.2–9.0] (36)
*C. leptum* subgroup	6.3 [6.2–6.3] (29)	NT	5.6 [5.5–5.7] (20)	6.6 [6.3–7.4] (36)	6.0 [5.6–6.3] (20)	8.3 [7.8–8.8] (27)	5.9 [5.7–6.5] (30)	8.1 [7.6–8.5] (27)
*Bacteroides fragilis* group	9.5 [8.7–9.6] (71)	NT	9.0 [8.5–9.6] (60)	9.4 [9.3–10.1] (45)	8.4 [7.0–9.3] (60)	9.3 [9.2–9.9] (45)	9.4 [8.6–9.6] (70)	9.1 [8.8–9.2] (45)
*Bifidobacterium*	7.0 [6.2–7.7] (42)	NT	9.5 [9.0–9.8]**^,^ *** (100)	9.0 [7.9–9.1] (27)	9.7 [9.4–10.2]**^,^ ***^,^ **** (100)	8.9 [8.8–9.6] (45)	9.7 [9.2–9.9]**^,^ *** (100)	9.2 [8.9–9.6] (45)
*Bifidobacterium breve* Yakult	<6.0 (0)	NT	9.1 [8.4–9.7]**^,^ *** (100)	<6.0 (0)	9.2 [8.6–9.5]**^,^ *** (100)	<6.0 (0)	9.0 [8.9–9.3]**^,^ *** (100)	<6.0 (0)
*Atopobium* cluster	7.9 [7.4–7.9] (42)	NT	6.2 [5.7–6.5] (30)	8.3 [8.1–8.4] (18)	6.9 [6.3–7.7] (30)	8.4 [8.2–8.6] (18)	6.7 [6.2–6.7] (30)	7.9 [7.5–8.2] (18)
*Prevotella*	6.2 [6.1–6.2] (29)	NT	5.4 [5.3–5.5] (20)	5.2 (9)	5.1 (10)	5.7 (9)	6.4 (10)	5.6 (9)
*C. perfringens*	<2.1 (0)	NT	2.9 (10)	<2.1 (0)	<2.1 (0)	<2.1 (0)	2.7 (10)	2.7 (9)
Facultative anaerobes								
Total *Lactobacillus*	5.0 [5.0–5.9] (57)	NT	5.4 [5.1–5.7] (20)	3.2 [2.9–3.3] (27)	4.3 [3.6–4.9] (60)	3.2 [2.7–3.3] (27)	4.7 [3.9–4.8] (60)	3.7 [3.6–3.8] (27)
*Enterobacteriaceae*	8.6 [8.2–8.7] (71)	NT	7.5 [7.2–7.8] (60)	8.0 [7.8–8.8] (73)	7.5 [7.1–8.0]* (60)	8.5 [8.3–9.2] (82)	7.7 [7.0–8.1]*^,^ **** (90)	9.4 [8.9–9.6]**** (91)
*Enterococcus*	8.7 [8.3–9.6] (71)	NT	8.2 [6.7–9.0] (70)	8.6 [8.2–9.5] (82)	8.4 [7.5–9.4]**** (100)	8.5 [8.2–8.8] (100)	8.3 [8.0–8.8] (100)	8.5 [7.9–9.5] (100)
*Staphylococcus*	8.1 [7.2–9.1] (71)	NT	6.9 [6.4–7.4] (90)	8.5 [6.3–9.0] (100)	6.2 [5.1–7.2]**** (80)	6.7 [5.5–7.2] (100)	6.3 [5.1–6.7]* (100)	7.2 [6.8–8.1] (100)
Obligate aerobes								
*Pseudomonas*	4.3 (14)	NT	<2.9 (0)	3.5 [3.1–3.6] (27)	3.4 (10)	3.5 [3.5–3.5] (18)	<2.9 (0)*	3.9 [3.7–4.5] (36)

Values are median log_10_ cells/g [IQR] (% detection rate). *NT* not tested. *p < 0.05, **p < 0.01 vs. group B, ***p < 0.05 vs. before initiating BBG, ****p < 0.05 vs. before surgery.

### Fecal organic acid concentration and pH

The total postoperative concentration of organic acids was significantly higher in group A than in group B and was significantly higher than the total concentration before initiating BBG-01 (Table 4). The significantly higher concentrations of acetic acid in group A than in group B both immediately and 1 week after surgery are particularly noteworthy (Table 4). Also, the concentration of acetic acid after initiating BBG-01 was significantly higher than that before initiating BBG-01. The fecal pH before initiating BBG-01 was significantly higher than that 1 week after surgery. Finally, the fecal pH tended to be lower in group A than in group B (before surgery *p* = 0.067, immediately after surgery *p* = 0.067, 1 week after surgery *p* = 0.053).

**Table 4:** Fecal organic acid concentrations and pH

			**Surgery**					
	**Before BBG**		**Before**		**Immediately after**		**1 week after**	
	**Group A**	**Group B**	**Group A**	**Group B**	**Group A**	**Group B**	**Group A**	**Group B**
	***n*** **= 7**		***n*** **= 10**	***n*** **= 11**	***n*** **= 10**	***n*** **= 11**	***n*** **= 10**	***n*** **= 11**
Total organic acids	21.7 [14.1–34.8] (100)	NT	52.6 [29.7–64.9] (100)	31.9 [26.2–62.9] (100)	61.3 [51.5–99.8]**^,^ *** (100)	40.7 [17.1–48.5] (100)	95.2 [61.0–105.4]*^,^ *** (100)	48.0 [39.9–88.6] (100)
Succinic acid	6.3 [4.9–8.2] (57)	NT	10.6 [8.6–14.2] (70)	16.2 [7.8–30.9] (73)	3.5 [1.3–15.0] (70)	11.2 [7.2–21.7] (45)	9.4 [1.9–15.2] (90)	3.3 [2.5–21.5] (64)
Lactic acid	10.0 [6.2–18.0] (57)	NT	7.8 [3.0–15.5] (70)	4.9 [4.2–7.1] (64)	8.9 [7.2–10.8] (90)	7.3 [5.5–8.3] (45)	18.8 [5.1–27.7] (100)	14.6 [3.8–24.2] (73)
Formic acid	3.6 [2.4–4.7] (29)	NT	2.6 [1.5–4.7] (60)	3.3 [2.5–6.4] (36)	5.4 [3.9–5.9] (60)	2.5 [1.5–3.4] (91)	2.2 [1.1–3.4] (50)	2.5 [1.9–3.8] (64)
Acetic acid	12.2 [7.3–21.7] (100)	NT	31.3 [22.6–46.4]*** (100)	20.1 [16.6–29.4] (100)	46.9 [41.0–69.0]**^,^ ***^,^ **** (100)	20.4 [10.3–31.0] (100)	53.8 [39.7–73.4]*^,^ *** (100)	36.7 [11.8–47.5] (100)
Propionic acid	0.9 (14)	NT	2.4 [2.0–5.0] (30)	4.4 [3.6–5.6] (36)	4.5 [2.7–6.2] (20)	3.1 [1.9–4.2] (18)	7.9 [4.5–7.9] (30)	13.3 [4.2–23.5] (36)
Butyric acid	<0.55 (0)	NT	<0.55 (0)	0.8 (9)	<0.55 (0)	3.6 [2.2–4.9] (18)	<0.55 (0)	2.9 [1.7–4.2] (18)
Isovaleric acid	<0.8 (0)	NT	<0.8 (0)	7.0 (9)	<0.8 (0)	<0.8 (0)	<0.8 (0)	2.7 (9)
Valeric acid	<0.65 (0)	NT	<0.65 (0)	<0.65 (0)	<0.65 (0)	<0.65 (0)	<0.65 (0)	<0.55 (0)
pH	6.7 [6.4–7.4] (100)		5.5 [5.3–6.0] (100)	6.3 [6.0–6.9] (100)	5.4 [5.3–6.4] (100)	6.4 [6.1–6.7] (100)	5.6 [5.5–5.6]*** (100)	6.0 [5.6–6.3] (100)

Values are median log_10_ cells/g [IQR] (% detection rate). *NT* not tested. *p < 0.05, **p < 0.01 vs. group B, ***p < 0.05 vs. before initiating BBG, ****p < 0.05 vs. before surgery.

## Discussion

The perioperative administration of probiotics to neonates undergoing cardiac surgery was well tolerated and significantly improved their fecal microbiota, expressed by a higher detection rate of obligate anaerobe and concentration of organic acids and a lower pH. The intestinal microbiota typically develops within 3 to 4 h after birth and stabilizes within approximately 2 weeks. Obligate anaerobes account for >95% of the commensal microbiota, with *Bifidobacterium* as the predominant microorganism [6]. As observed in this study, the microbiota in neonates undergoing cardiac surgery may be disrupted below the detection limit throughout the perioperative period. By contrast, the administration of *Bifidobacterium* may successfully preserve the intestinal microbiota and decrease the presence of pathogenic *Enterobacteriaceae* significantly. This is concordant with previous studies, which found a decrease in *Enterobacteriaceae* after the administration of BBG-01 to Bangladeshi children <5 years of age [17]. The concentration of acetic acid, one of the short-chain fatty acids, was also significantly higher in recipients of BBG-01. Short-chain fatty acids are important anions in the colonic lumen, which influence both the morphology and function of the colonocytes, and their increase lowers the pH, which indirectly modifies the composition of the colonic microbiota and increases the absorption of minerals [20]. Shin et al. have suggested that a low pH is important to decrease the incidence of infections due to the O157 subtype of *Escherichia coli*[21]. These changes in the intestinal environment might contribute to the trend of earlier postoperative recovery of enteral nutritional intake and defecation. A recent clinical study has also shown that perioperative administration of synbiotics in esophageal surgery facilitated enteral nutrition [22]. An earlier recovery of intestinal function is favorable, as it might prevent the malnutrition frequently observed in neonates presenting with congenital heart disease [5].

We used a *B. breve* strain Yakult as the probiotic, which is naturally resistant to cefazolin, an antimicrobial often used for surgical prophylaxis, and obtained significant recovery in fecal samples. Moreover, several clinical studies have shown its beneficial effects in children [15-17]. Kitajima et al. found that the early administration of *B. breve* significantly improved the function of the digestive tract and promoted weight gain in neonates [15]. Wada et al. observed a lower incidence of fever and fewer days of parenteral antimicrobial therapy in patients undergoing chemotherapy for pediatric malignancies [16]. Those indicate that the use of *B. breve* strain Yakult could be a possible option for perioperative use of neonatal heart surgery.

### Limitations

The cardiac anomaly tended to differ among the groups. Group B contains more arch anomalies including interrupted aortic arch or hypoplastic left heart syndrome. Since arch anomalies might tend to cause intestinal ischemia and associated clinical morbidities, this difference might be contributed to clinical outcomes, rather than the administration of probiotics. The risk-adjusted congenital heart surgery (RACHS) categories, which represent surgical complexity and associated outcomes, however, were similar between the groups (Table 1).

## Conclusions

While our observations suggest improvements in gut function conferred by the administration of *B. breve*, it was insufficient to draw any significant conclusions regarding its effect on the incidence of postoperative complications including infection or postoperative recovery. This might be inherent due to the nature of pilot study with small sample size. The clinical benefits of this intervention remain to be confirmed in larger, future trials.

## Competing interests

The authors declare that they have no competing interests.

## Authors’ contributions

TU carried out the clinical data acquisition, performed the statistical analysis of clinical data, and drafted the manuscript. NS conceived the study, participated in its design and coordination, and helped draft the manuscript. TA and KN carried out the fecal examination and performed the statistical analysis of fecal examination. TI participated in the design of the study and revised it critically for important intellectual content. All authors read and approved the final manuscript.
